# A Novel Microduplication Spanning Exons 8–16 of *ATP2C1* That Was Undetectable by Standard Sanger Sequencing in a Japanese Patient With Hailey–Hailey Disease

**DOI:** 10.3389/fmed.2020.00492

**Published:** 2020-09-04

**Authors:** Kwesi Teye, Hiroshi Koga, Takahiro Hamada, Mitsuhiro Matsuda, Mikio Ichiki, Sanae Numata, Norito Ishii, Takekuni Nakama

**Affiliations:** ^1^Kurume University Institute of Cutaneous Cell Biology, Kurume, Japan; ^2^Department of Dermatology, Kurume University School of Medicine, Kurume, Japan

**Keywords:** Hailey–Hailey disease, microduplication, *ATP2C1*, SPCA1, MLPA, genetic disease, Japan

## Abstract

Hailey–Hailey disease (HHD) is genetic skin disorder characterized by repeated and exacerbated skin lesions in friction regions. *ATP2C1*, encoding SPCA1, was demonstrated to be the responsible gene for HHD pathogenesis. However, for some cases, no *ATP2C1* mutation could be determined by standard Sanger sequencing, thereby obscuring the cause and diagnosis of HHD. In this study, we investigated the possibility that HHD is caused by complex *ATP2C1* defects using multiplex ligation-dependent probe amplification (MLPA) analysis for 10 of 50 cases in our institute without *ATP2C1* mutations. In one female Japanese patient and her father, who also show HHD, MLPA followed by polymerase chain reaction (PCR) analyses revealed a novel duplication of exons 8–16 of *ATP2C1*. The duplication was predicted to add 20,615 base pairs, 882 nt, and 294-amino-acid residues to the genome, mRNA and SPCA1 protein, respectively. By reverse transcriptase–PCR using patient skin RNA, we could confirm that a larger transcript was produced and we found that the abundance of the normal transcript was clearly reduced in the patient. Putative structures of wild-type and duplicated proteins revealed differences in arrangement of SPCA1 domains that may have functional consequences. Strikingly, the phosphorylation and the nucleotide-binding domains were interrupted by insertion of a partial actuator, transmembrane, and phosphorylation domains. The additional 294 amino acids appear to disrupt SPCA1 structure and function, causing HHD. Our study expands the spectrum of genetic defects in HHD and showed that disruption of SPCA1 structure and function by the microduplication caused HHD in the patient and her father.

## Introduction

Hailey–Hailey disease (HHD, OMIM 169600) is genetic skin disorder characterized by repeated and exacerbated skin lesions in friction regions such as the neck, axillae, groin, and perineum (1–4). HHD generally presents between 2 and 4 decades of life (1). The incidence of HHD is about 1:50,000–1:40,000 in various populations and affects both genders equally (5).

HHD is characterized by abnormal keratinocyte adhesion in the suprabasal layers of the epidermis (6). Genetic studies have revealed that mutations in ATPase calcium-transporting type 2C member 1 gene (*ATP2C1*), which encodes a calcium pump protein, secretory pathway Ca^2+^/Mn^2+^-ATPase protein 1 (SPCA1), are responsible for the pathogenesis of HHD (7, 8). HHD shows dominant mode of inheritance and haploinsufficiency of *ATP2C1* is recognized as a prevalent mechanism for dominant inheritance of HHD (7–9). To date, more than 150 mutations in *ATP2C1* have been identified in HHD (9, 10). However, for a group of patients with typical HHD, *ATP2C1* mutations could not be determined, thereby obscuring the cause and diagnosis of HHD in such cases (9, 11, 12). Also, in our ongoing analysis, no *ATP2C1* mutation could be found by standard Sanger sequencing in 10 of 50 cases, suggesting involvement of complex genomic defects in *ATP2C1* in the pathogenesis of HHD. Identification of disease-causing mutation is important for the patient and the clinician and can help to tailor treatments to improve the quality of life of the patients. In this study, we investigated the possibility that HHD is caused by complex defects in *ATP2C1* in these 10 patients by using multiplex ligation-dependent probe amplification (MLPA) analysis, which has been shown to be useful for detecting gene copy number variation (CNV) in various conditions and diseases (13–19).

## Materials and Methods

### Patients

Ten of 50 patients with symptoms of HHD without *ATP2C1* mutation performed by Sanger sequencing were enrolled in this study. Diagnosis of HHD was performed by dermatologists based on clinical and histopathological features as described previously (12). All studies were performed in compliance with Helsinki principles.

### MLPA Analysis

MLPA was performed as describe previously (16). However, probes were selected manually because the probe design tool failed to return any valid probe. Even-numbered exons of *ATP2C1* were analyzed by MLPA. Hybridization sequences of the MLPA probes are provided in the Supplementary Table 1.

### Polymerase Chain Reaction Analysis to Determine Duplication Breakpoint

After MLPA analysis, polymerase chain reaction (PCR) was performed with *ATP2C1* exon 16 or 17 forward primers and exon 7 or 8 reverse primers. Subsequently, PCR was performed with several forward primers located in intron 16 and exon 8 reverse primer. The sequences of the forward primers are 5′-TATTTTAAGCCGCCACCTTG-3′ (0485), 5′-CCTGGCACAAGTGATCTTCC-3′ (1088), 5′-AGGCGATTTTCAGCTGATGT-3′ (1679), 5′-TTGAAGGCAGGGAGTAAGG-3′ (2253), 5′-TTTGTGGCTGGGTTCCTAAC-3′ (2858), 5′-GAAAGCCAAAGACCAAACCA-3′ (3492), 5′-GATGCCTGAAACCATGAAT-3′ (4059), 5′-CAGCAGGCCTAGAAATCAGC-3′ (4608), and 5′-GGAAATGGGGAAATTAACA-3′ (5280). Numbers indicate the number of base pairs (bp) from the end of exon 16. The sequence of exon 8 reverse primer is 5′-GGACACAGATTCTCCCCAGT-3′, and that of exon 16 forward primer is 5′-CCTAGTTACAAGTGGTGACC-3′ (0). PCR was performed with PrimestarMax PCR Mastermix (Takara, Ohtsu, Japan). The PCR reaction consisted of initial denaturation at 94°C for 2 min followed by 35 cycles of denaturation for 10 s at 98°C, annealing for 10 s at 60°C, and extension for 1 min at 72°C. PCR products were purified and sequenced directly or cloned using Zero Blunt TOPO PCR Cloning Kit (Thermo Fisher Scientific, Inc., Rockford, IL, USA) before sequencing.

### Development of Multiplex PCR to Detect Duplication

A multiplex PCR method was developed by using a common forward primer located in intron 16 and two allele-specific reverse primers located in intron 16 or intron 7 after the breakpoint. Wild-type and mutant were expected to produce 1.65- and 1.1-kb PCR products, respectively. The sequences of the primers are 5′-TCTCATCACCCTACAGCAAGGC-3′ (forward), 5′-CATTTCTAATTGGCTGATTTCTAGGC-3′ (wild-type reverse), and 5′-ACAGCAAAGCTTCTGAAAGGAGG-3′ (mutant reverse). PCR was performed with KOD one PCR Mastermix (Toyobo, Osaka, Japan). The PCR reaction consisted of initial denaturation at 94°C for 2 min followed by 35 cycles of denaturation for 10 s at 98°C, annealing for 5 s at 60°C, and extension for 5 s at 68°C.

### RNA Isolation, RT-PCR, Cloning, and DNA Sequencing

Total RNA was isolated from punch skin biopsy of patient using RNeasy protect Kit (Qiagen) and converted to cDNA using Superscript RT Kit (Thermo Fisher Scientific). PCR was performed with forward (located in exon 7) and reverse (located in exon 17) primers. PCR was also performed with forward and reverse primers located in exons 15 and 9, respectively. Some PCR products were purified and sequenced directly or cloned using Zero Blunt TOPO PCR Cloning Kit (Thermo Fisher Scientific) before sequencing.

### Bioinformatics

Genomic DNA sequence information was obtained from UCSC genome browser and tools (20–22). Primers were designed with Primer-Blast (23) and Primer3 (24, 25). Repetitive DNA elements were analyzed with Dfam (26).

## Results

### Identification of a Novel *ATP2C1* Duplication in an HHD Patient

**Figure 1:**
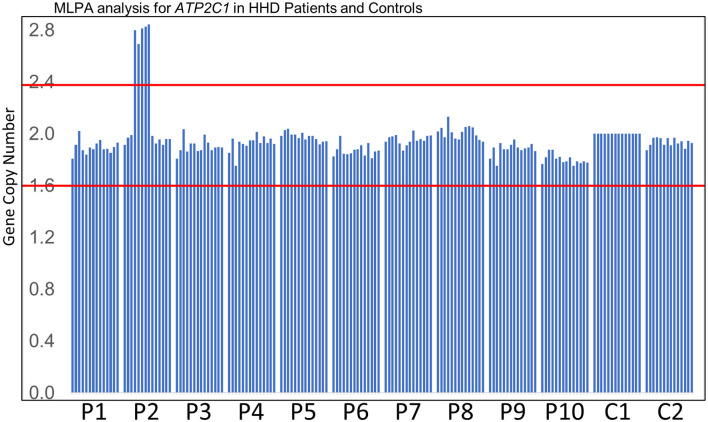
MLPA analysis. Results of MLPA analysis of even-numbered exons of *ATP2C1* for 10 patients (P1–P10) who did not disclose any *ATP2C1* mutation by Sanger sequencing and two normal controls (C1–C2). C1 was used for normalization. P2 showed abnormal gene copy number of some exons. Gene copy number between the red lines indicates the normal MLPA range.

**Figure 2:**
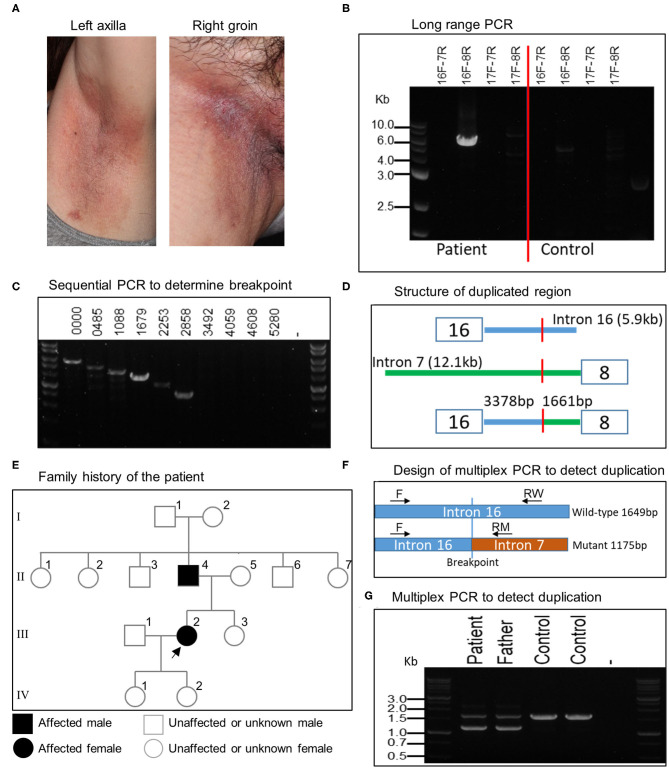
Clinical features of patient and characterization of large *ATP2C1* duplication in HHD. **(A)** Clinical features of HHD patient with *ATP2C1* duplication showing large macerated plaques on the left axilla and right groin. **(B)** Long-range PCR analysis of *ATP2C1* with various primer combinations. **(C)** PCR analysis with forward primers spanning intron 16 and exon 8 reverse primer. Numbers indicate bp from the end of exon 16. **(D)** Schematic structure of the duplicated region. **(E)** Family history of the affected patient. The patient and her father were examined in this study. Alive or deceased were not indicated. Children of the father's siblings were not indicated. **(F)** Development of multiplex PCR method for duplication detection. Common forward **(F)** and two allele-specific reverse RW (wild-type) and RM (mutant) primers were used for PCR. **(G)** Detection of *ATP2C1* duplication using multiplex PCR.

MLPA analysis of even-numbered exons of *ATP2C1* revealed abnormal gene copy number of some exons in one patient (Figure 1). The analysis suggested that at least exons 8–16 (of 28 exons) are duplicated in the patient (Figure 1). In MLPA analysis, gene copy number (dosage quotient multiplied by 2) between 1.6 and 2.4 is considered normal, 0 is considered homozygous deletion, between 0.8 and 1.3 is considered heterozygous deletion, between 2.6 and 3.3 is considered heterozygous duplication, and between 3.5 and 4.3 is considered homozygous duplication, as suggested by the MLPA manufacturer (MRC Holland, Amsterdam, the Netherlands). Because the gene copy numbers of the duplicated exons in our analysis are around 2.8 (Figure 1), the duplication is considered to be in heterozygous state. The patient is a 60-year-old Japanese woman who shows large macerated plaques on the axilla and groin areas (Figure 2A). Her condition is relatively mild. Because only even-numbered exons were examined, we designed and performed PCR to determine structure of the duplicated region using *ATP2C1* exon 16 or 17 forward primers with exon 7 or 8 reverse primers. Previous studies have shown that most duplications appear to be tandem and in the direct orientation (27). The choice of the primer combination is consistent with tandem duplication in the direct orientation, which we assumed by default to begin our investigation. We found that a combination of exon 16 forward and exon 8 reverse primers produced prominent PCR band of about 6 kb in the patient but not in the control (Figure 2B), confirming that exons 8–16 are duplicated.

### Determination of Duplication Breakpoint and Detection of Duplication by Multiplex PCR

To determine the breakpoint, we performed PCR with exon 8 reverse primer and several forward primers spanning about 6 kb of intron 16 in decrement of 505–634 bp using genomic DNA (Figure 2C). The last forward primer in intron 16 producing a band with exon 8 reverse primer was at position 2,858 bp from the end of exon 16 (Figure 2C). The PCR product was cloned and sequenced. Alignment of the resultant sequence revealed that the breakpoint was located 3,378 bp after exon 16 and 1,661 bp before exon 8 (Figure 2D). The duplication size was calculated to be 20,615 bp and designated as c.532-1661_1413+3378dup. According to the patient, her father, but none of her children, showed HHD phenotype (Figure 2E). It is not clear whether other members in the family history (Figure 2E) are affected. We then developed a multiplex PCR method for detecting this duplication (Figure 2F). By using this PCR, we detected the duplication also in the patient's father (Figure 2G), who also show HHD phenotype. The duplication was absent in 2 controls (Figure 2G) and 41 other normal controls (data not shown). The other members in the family were neither examined nor tested for the duplication in this study.

### Duplication Leads to Production of Larger In-Frame Transcript in the Patient

**Figure 3:**
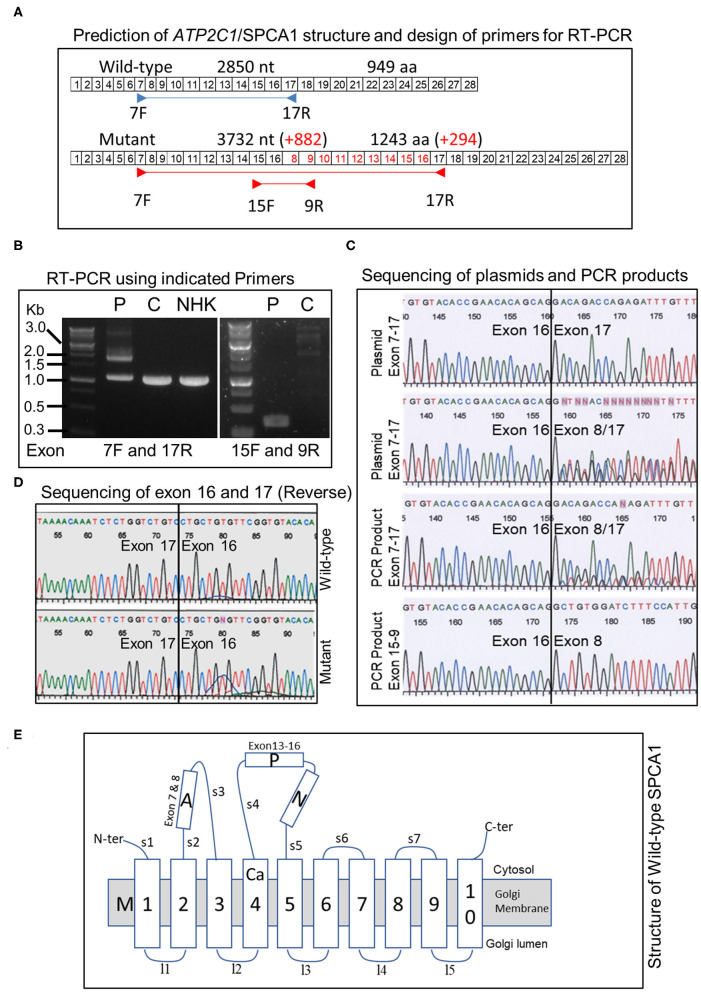
Analysis of *ATP2C1* mRNA in HHD and controls and prediction of protein structures. **(A)** Prediction of structure of transcript and proteins and design of primers for RT-PCR. Boxed numbers indicate exon numbers and primer positions are indicated. **(B)** RT-PCR analysis of mRNA from patient, normal skin, and NHK. PCR was performed with primers located in exon 7 forward and exon 17 reverse (left) or exon 15 forward and exon 9 reverse (right). P, patient; C, control; NHK, normal human keratinocytes. **(C)** Sequence analysis of PCR products and plasmids with cloned PCR products. **(D)** Sequence analysis between exons 16 and 17 of wild-type and mutant *ATP2C1* mRNA. **(E)** Schematic structure of wild-type SPCA1 protein. The Ca^2+^ binding site is located in M4. A, actuator; P, phosphorylation; N, nucleotide-binding; and M, transmembrane domains.

The duplication is predicted to add 882 nt to the transcript (Figure 3A). To verify this, we performed RT-PCR using patient's skin RNA (Figure 3A). Using primers located in exon 7 forward and exon 17 reverse, we obtained two major bands in the patient, whereas only one band, corresponding to the normal transcript was obtained in control skin and cultured normal human keratinocytes (Figure 3B). The results also showed that the abundance of the normal transcript is clearly reduced in the patient (Figure 3B). PCR with exon 15 forward and exon 9 reverse primers produced a single band of about 350 bp in the patient but not in the control (Figure 3B). Cloning and sequencing of the plasmids and PCR products confirmed that exon 8 was transcribed after exon 16 in the duplicated region (Figure 3C), and the sequence between exons 16 and 17 is normal in the mutant (Figure 3D). Remarkably, the larger transcript with additional 882 nt is in-frame and predicted to produce a protein with 294 extra amino acids (Figure 3A).

### Structural Variation Between Wild-Type and Duplicated SPCA1 Proteins

Putative structures of wild-type and duplicated proteins revealed differences in arrangement of SPCA1 domains (Figure 3E). The protein structure was described previously (9). Strikingly, the phosphorylation (P) and nucleotide-binding (N) domains may be interrupted by insertion of partial actuator (A), transmembrane (M), and P domains, based on the structure of the wild type. Although a seemingly normal arrangement of A-P-N may be maintained in the mutant, part of the A domain (exon 7) is missing (Figure 3E).

### Mechanism Underlying Duplication

**Figure 4:**
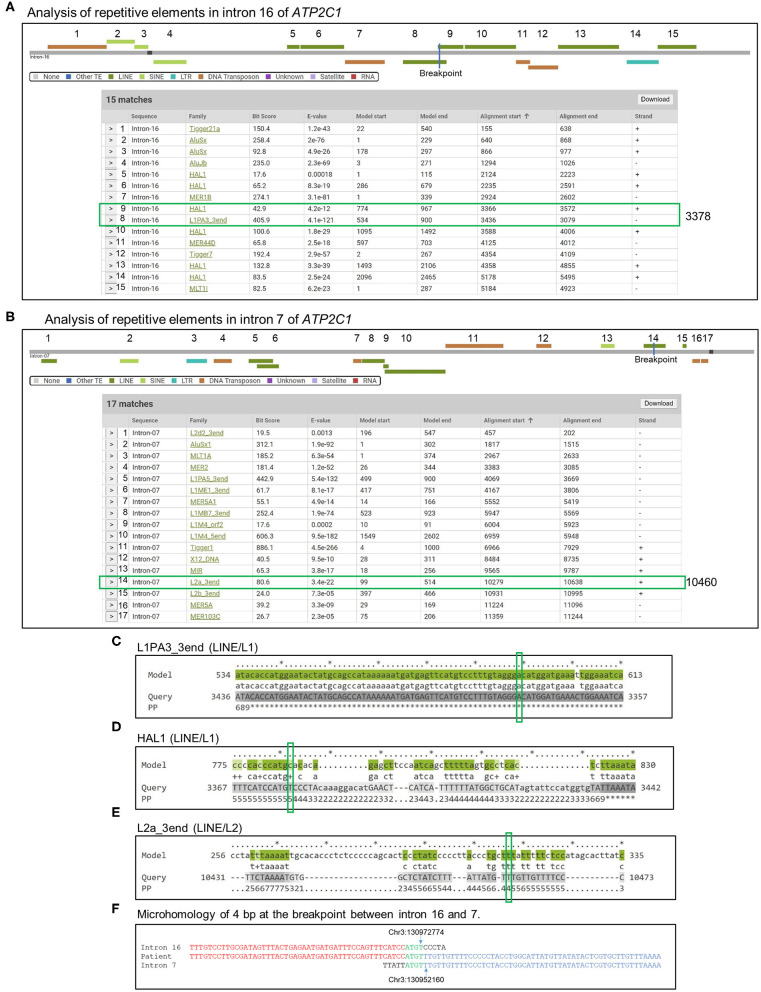
Analysis of mechanism of duplication. **(A,B)** Introns 16 **(A)** and 7 **(B)** sequences were analyzed by Dfam to detect repetitive DNA sequences that might mediate the duplication. Detected repetitive DNA elements are shown. Breakpoints are indicated by blue lines in the scheme, and positions are indicated by green boxes in the table. **(C–E)** Alignment of model sequences that located in the breakpoint against introns 16 and 7 to determine sequence homologies around the breakpoint. Breakpoints are indicated by green boxes. PP, posterior probability or degree of confidence at each position. *Highest confidence and low numbers indicate low confidence. **(F)** Identification of microhomology sequences of 4 bp at the breakpoint between introns 16 and 7.

We screened for repetitive DNA elements in introns 16 and 7 of *ATP2C1* using Dfam database (26). More than 30 elements were found (Figures 4A–C). Three of the elements were located in the breakpoint (Figures 4A,B). Further analysis indicated that the long interspersed nuclear element (LINE) L1 of L1PA3_3end type has very low e-value and high bit score (Figure 4A) and showed highest homology against the model (Figures 4C–E), suggesting that it is likely to be responsible for initiation of the duplication. Identification of homology of only 4 bp at the breakpoint between introns 16 and 7 (Figure 4F) suggested that the initial break was repaired by a nonhomology end joining (NHEJ) mechanism, which requires up to 4-bp microhomology (28–30).

## Discussion

Current methods for routine mutation detection rely on PCR and direct DNA sequencing and are limited to a specific gene region. It is apparent that this type of analysis cannot detect all types of genetic defects underlying various diseases. By employing MLPA, which is simple, sensitive, and cost-effective for CNV analysis, we were able to detect a large and novel duplication in *ATP2C1* in a family with HHD, which to the best of our knowledge was previously unreported. The duplication spans exons 8–16 and adds 882 nt to the transcript and 294 amino acids to the protein, which is in-frame. The duplication was likely caused by L1PA3_3end LINE/L1 repetitive DNA element and repaired by an NHEJ mechanism. We successfully developed a novel one-step multiplex PCR method, which was useful for the sensitive and specific detection of this duplication.

The protein encoded by *ATP2C1*, SPCA1, which is located on the Golgi apparatus, plays an important role in epidermal cell–cell adhesion and differentiation, by actively transporting Ca^2+^ from the cytoplasm into the Golgi apparatus (31). Ca^2+^ transport cycle involves phosphorylation and dephosphorylation of SPCA1 and utilization of ATP (32). During the reaction cycle, Ca^2+^ binding in M4 domain causes phosphorylation of P domain of SPCA1, and movement of A domain causes rearrangement of several domains leading to opening of the lumen gate for release of Ca^2+^ and dephosphorylation by A domain makes SPCA1 regain affinity for Ca^2+^ (32). Regulation of calcium, which plays an important role in cell adhesion, was found to be impaired in the cytoplasm of cultured keratinocytes from HHD patients, underscoring the importance of calcium control in normal function of stratified squamous epithelia (7). One limitation of our study is the absence of protein analysis in the patient, because we were unable to obtain sufficient skin material to perform protein analysis. It is possible that the mutant may not be produced in the HHD cells. However, successful detection of the mutant mRNA and the mild condition may suggest production of a mutant protein with reduced function. Putative structure of mutant SPCA1 suggested that the functional A domain may not be in optimal proximity to the P and N domains to control their phosphorylation and dephosphorylation, respectively. Alternatively, the partial A domain may not be efficient in controlling phosphorylation and dephosphorylation of SPCA1. Either way, the additional 294-amino-acid residues appear to disrupt SPCA1 structure and function, including Ca^2+^ binding by inclusion of additional M4 domain and probably impaired localization to the Golgi apparatus, causing relatively mild HHD. Thus, absence or reduced function of the mutant protein may lead to HHD through a haploinsufficiency mechanism, which is a hallmark in HHD. However, it should be noted that modifying genes and/or environmental factors may affect intrafamilial and interfamilial manifestations of HHD with this duplication (12).

Importantly, using our novel multiplex PCR method, we identified the same duplication in the patient's father, who also show HHD, but not in 43 normal controls, suggesting that the duplication segregates with HHD. To the best of our knowledge, this is first report of multiple exon duplication within *ATP2C1* in HHD. It will be interesting to determine whether the same duplication could be found in other countries besides Japan. Our PCR method will provide a one-step method for analyzing this duplication to provide definitive genotype–phenotype correlation for this duplication in HHD.

## Conclusion

We identified a novel microduplication spanning exons 8–16 of *ATP2C1* in a Japanese family with HHD phenotype. Our study expands the spectrum of genetic defects in HHD and showed that disruption of structure and function of SPCA1 by this novel and large duplication is the genetic cause of HHD in the patient and her father. While MLPA was useful for detecting this duplication, which could not be detected by standard Sanger sequencing, whole- or targeted-exome sequencing, which are well-established methods for candidate gene and CNV analyses (33–35), may be needed to detect the pathogenic defects in the other HHD cases without *ATP2C1* defects.

## Data Availability Statement

All datasets generated for this study are included in the article/Supplementary Material.

## Ethics Statement

The studies involving human participants were reviewed and approved by Medical Ethics Committee of Kurume University School of Medicine. The patients/participants provided their written informed consent to participate in this study.

## Author Contributions

KT, HK, and TH designed the study and wrote the manuscript. All authors performed acquisition, analysis or interpretation of data for the manuscript, revised the manuscript, and provided approval for publication of the content.

## Conflict of Interest

The authors declare that the research was conducted in the absence of any commercial or financial relationships that could be construed as a potential conflict of interest.
