# Supplementary material for: A Novel Microduplication Spanning Exons 8–16 of ATP2C1 That Was Undetectable by Standard Sanger Sequencing in a Japanese Patient With Hailey–Hailey Disease
Source: Front Med (Lausanne). 2020 Sep 4;7:492. doi: 10.3389/fmed.2020.00492 (PMC7498616; doi:10.3389/fmed.2020.00492)
Supplement: Supplementary file 1 [file Data_Sheet_1.PDF]

Table S1. Hybridization sequences of probes used for MLPA analysis of *ATP2C1* in HHD

ATP2C1 Exon Number	Left Hybridisation Sequence	Right Hybridisation Sequence	Product Size (bp)*
2	GGTGAAAATGAGACAATGATTCCTGTATTGA	CATCAAAAAAAGCAAGTGAATTACCAGTCAG	104
4	TTCTGGCTTCTGCAGTCATCAGTGTTTTAAT	GCATCAGTTTGATGATGCCGTCAGTATCACT	108
6	GGAATATCGTTCAGAAAAATCTCTTGAAGAA	TTGAGTAAACTTGTGCCACCAGAATGCCATT	112
8	GGAGATCTTGCATCGAGAAGTAACATTGCCT	TTATGGGAACACTGGTCAGATGTGGCAAAGC	116
10	TCAAGGCACCAAAAACCCCTCTGCAGAAGAG	CATGGACCTCTTAGGAAAACAACTTTCCTTT	120
12	TCTCCCCATTGTGGTCACAGTGACGCTAGCT	CTTGGTGTTATGAGAATGGTGAAGAAAAGGG	124
14	ATCAATTTGGGGAAGTGATTGTTGATGGTGA	TGTTGTTCATGGATTCTATAACCCAGCTGTT	128
16	CAGAAAAGCTGAATACCCTTTTAGCTCTGAG	CAAAAGTGGATGGCTGTTAAGTGTGTACACC	132
18	CTGAACTGGGACAGCTGACATTTCTTGGCTT	GGTGGGAATCATTGATCCACCTAGAACTGGT	136
20	CAGGTTGCAGTATTTTACAGAGCTAGCCCAA	GGCACAAGATGAAAATTATTAAGGTGAGTGT	140
22	GCAATCGAAGAGGGTAAAGGGATTTATAATA	ACATTAAAAATTTCGTTAGATTCCAGCTGAG	144
24	GAACCAGTGGATAAAGATGTCATTCGTAAAC	CTCCTCGCAACTGGAAAGACAGCATTTTGAC	148
26	GGACTCTGCAGTAATAGAATGTTTTGCTATG	CAGTTCTTGGATCCATCATGGGACAATTACT	152
28	GGAGTGGACAGCAGCTGGTTGAGATACATCC	CCATCTGGAGACAGGACTGCCACTGACAGAA	156

*Product size includes the sequences of hybridization (31 bp each), stuffer (variable, 0-26 bp each) and MLPA primers (19 and 23 bp)
